# Behavior Change Approaches in Digital Technology–Based Physical Rehabilitation Interventions Following Stroke: Scoping Review

**DOI:** 10.2196/48725

**Published:** 2024-04-24

**Authors:** Helen J Gooch, Kathryn A Jarvis, Rachel C Stockley

**Affiliations:** 1 Stroke Research Team School of Nursing and Midwifery University of Central Lancashire Preston United Kingdom

**Keywords:** behavior change, behavior therapy, digital health technology, digital health, digital technology, health behavior, physical and rehabilitation medicine, scoping review, stroke rehabilitation

## Abstract

**Background:**

Digital health technologies (DHTs) are increasingly used in physical stroke rehabilitation to support individuals in successfully engaging with the frequent, intensive, and lengthy activities required to optimize recovery. Despite this, little is known about behavior change within these interventions.

**Objective:**

This scoping review aimed to identify if and how behavior change approaches (ie, theories, models, frameworks, and techniques to influence behavior) are incorporated within physical stroke rehabilitation interventions that include a DHT.

**Methods:**

Databases (Embase, MEDLINE, PsycINFO, CINAHL, Cochrane Library, and AMED) were searched using keywords relating to behavior change, DHT, physical rehabilitation, and stroke. The results were independently screened by 2 reviewers. Sources were included if they reported a completed primary research study in which a behavior change approach could be identified within a physical stroke rehabilitation intervention that included a DHT. Data, including the study design, DHT used, and behavior change approaches, were charted. Specific behavior change techniques were coded to the behavior change technique taxonomy version 1 (BCTTv1).

**Results:**

From a total of 1973 identified sources, 103 (5%) studies were included for data charting. The most common reason for exclusion at full-text screening was the absence of an explicit approach to behavior change (165/245, 67%). Almost half (45/103, 44%) of the included studies were described as pilot or feasibility studies. Virtual reality was the most frequently identified DHT type (58/103, 56%), and almost two-thirds (65/103, 63%) of studies focused on upper limb rehabilitation. Only a limited number of studies (18/103, 17%) included a theory, model, or framework for behavior change. The most frequently used BCTTv1 clusters were feedback and monitoring (88/103, 85%), reward and threat (56/103, 54%), goals and planning (33/103, 32%), and shaping knowledge (33/103, 32%). Relationships between feedback and monitoring and reward and threat were identified using a relationship map, with prominent use of both of these clusters in interventions that included virtual reality.

**Conclusions:**

Despite an assumption that DHTs can promote engagement in rehabilitation, this scoping review demonstrates that very few studies of physical stroke rehabilitation that include a DHT overtly used any form of behavior change approach. From those studies that did consider behavior change, most did not report a robust underpinning theory. Future development and research need to explicitly articulate how including DHTs within an intervention may support the behavior change required for optimal engagement in physical rehabilitation following stroke, as well as establish their effectiveness. This understanding is likely to support the realization of the transformative potential of DHTs in stroke rehabilitation.

## Introduction

### Background

Digital health technologies (DHTs) comprise apps, programs, or software used in the health and social care systems [1]. They are considered to have almost unlimited potential to transform health care interventions and delivery and empower people to take a greater role in their own care and well-being [2,3].

Stroke is one of the leading causes of acquired disability worldwide, with around 12 million people experiencing a stroke each year [4]. Rehabilitation is a complex, multifaceted process [5] that facilitates those with health conditions and disabilities to participate in and gain independence in meaningful life roles [6]. It is considered an essential aspect of health care provision following a stroke [7] as a means to address poststroke impairments, which can involve motor, sensory, and cognitive functions. Changes in the ability to move due to impairment of both movement and sensory function are commonly experienced by people following a stroke [8] and are addressed by physical rehabilitation comprising regular, intensive practice and repetition of movements and tasks [9,10]. Conventional physical rehabilitation often struggles to deliver the intensity required to optimize recovery [11], and over recent years, there has been significant interest in the use of DHTs, such as virtual reality (VR), telerehabilitation, robotics, and activity monitors [12-16], to enhance and increase the intensity of rehabilitation. DHTs can provide a whole intervention or be used as a component of a wider intervention; the term DHT-based intervention has been used within this review to refer to both situations.

For many people who survive a stroke, rehabilitation requires individuals to engage in regular and frequent rehabilitative activities to achieve improvements in function and realize their optimal recovery. This necessitates adjustments to an individual’s behavior [17] over a sustained period of time. Changing behavior is a complex process and is underpinned by a variety of different theories, models, and frameworks [18], such as social cognitive theory [19] or the behavior change wheel framework [20]. Individual activities within a complex intervention that are designed to change behavior can be separated into replicable active components widely referred to as behavior change techniques (BCTs) [21]. Historically, labels applied to BCTs have lacked consensus, resulting in uncertainty and difficulty in comparing interventions. This has been addressed in the behavior change technique taxonomy version 1 (BCTTv1) [22], a classification system of 93 distinct BCTs clustered into 16 groups, which is a well-recognized tool to provide consistency with BCT reporting in interventions. DHTs provide an emerging opportunity to support the behavior change required within physical stroke rehabilitation interventions through facilitators that are embedded within the technology itself that aim to form, alter, or reinforce behaviors [23]. Understanding of this area is limited, with most literature exploring the use of DHTs to support behavior change focused on specific health-related behaviors such as physical activity or healthy eating [24] rather than as a core component of a type of rehabilitation intervention. Motivation is acknowledged to play an integral role in behavior change [25], and it is often assumed that DHTs provide motivation to engage with rehabilitation [26]. However, for this assumption to be realized, the DHTs must be able to support and deliver interventions that facilitate the vital changes in behavior needed to promote prolonged and sustained engagement in stroke rehabilitative activities. Imperative to this is understanding the theories, models, and frameworks that underpin interventions and the BCTs (active components) within the interventions [27-29]. The theories, models, and frameworks alongside the BCTs will be referred to hereinafter as approaches. Within the context of DHT-based physical stroke rehabilitation interventions, approaches to behavior change warrant further investigation.

### Aim and Objectives

This scoping review aimed to identify if and how behavior change approaches are incorporated within DHT-based physical stroke rehabilitation interventions. Specifically, it sought to:

1. Establish if behavior change theories, models, and frameworks, or BCTs, are described when reporting on DHT-based interventions that have been developed or evaluated for use in poststroke physical rehabilitation.
2. Identify if behavior change theories, models, or frameworks underpin the interventions and which of these are being used.
3. Identify if the BCTTv1 is being used to report BCTs within interventions.
4. Determine which BCTs (based on the BCTTv1) can be identified within the interventions.
5. Explore whether the type of technology influences the techniques used to change behaviors.

## Methods

### Review Methodology

A scoping review was completed and reported following established guidelines [30,31] and the Preferred Reporting of Systematic Reviews and Meta-Analyses Extension for Scoping Reviews (PRISMA-ScR; Multimedia Appendix 1) [32]. The protocol was registered with the Open Science Framework [33].

### Eligibility Criteria

Any published sources that reported a completed primary research study in which a behavior change approach could be identified within a DHT-based physical stroke rehabilitation intervention were included (Multimedia Appendix 2). Physical rehabilitation comprised interventions that addressed an impairment, or sequela of impairment, of sensory function and pain, neuromusculoskeletal and movement-related functions, or voice and speech, as defined by the International Classification of Functioning, Disability, and Health [34]. Completed primary research included all types of studies, both quantitative and qualitative, and no minimum sample size or intervention length was set. The BCTTv1 [22] was used to support the identification of BCTs within the interventions.

### Information Sources and Search Strategy

A systematic database search was conducted in Embase, MEDLINE, PsycINFO, CINAHL, Cochrane Database of Systematic Reviews, CENTRAL Register of Controlled Trials, and AMED on March 21, 2023. The search was completed in collaboration with an information specialist who provided support with the development of the free text and thesaurus search terms, created the final search, adjusted the searches for the different databases, and ran the search. It consisted of 4 distinct search streams: behavior change, DHT, physical rehabilitation, and stroke, which were then combined (Multimedia Appendix 3). Searches were restricted to the English language (due to review resources) and by date to search from 2001; the date restriction acknowledges the main time period of DHT growth [35], captures sources reported in systematic reviews of DHTs in stroke rehabilitation [12-16], and is reflected in other scoping literature exploring DHTs [24]. Additional sources were identified by hand searching, including scrutiny of the included source reference lists.

### Selection of Sources of Evidence

The titles and abstracts of deduplicated sources from database searches and hand searches were independently screened by 2 reviewers, 1 of whom had completed the BCTTv1 web-based training package [36] to inform decisions made around the use of BCTs. Any conflicts were discussed, and if a consensus was not reached, the source was included for full-text screening. Attempts were made to locate a completed study publication from eligible conference abstracts, protocols, and trial registry entries. Full-text sources were screened independently by 2 reviewers, and disagreements were resolved by a third reviewer. Reasons for full-text exclusion were recorded. EndNote X9 software (Clarivate) and the Rayyan web tool software (Qatar Computing Research Institute) [37] were used to facilitate the source selection process.

### Data Charting Process

A review-specific data charting tool was developed and initially piloted using 3 sources by 3 reviewers, and then further developed iteratively throughout the process [30]. Data charting was completed collectively by 2 reviewers. When several sources referred to a single study, these sources were grouped together for data charting, and if a source identified additional sources for further detail of the intervention (eg, a protocol or supplementary material), then this information was also used to support data charting.

### Data Items

The data charting tool was developed with reference to the Template for Intervention Description and Replication (TIDieR) checklist [27] and with a focus on the DHT-based intervention and behavior change approaches (Multimedia Appendix 4 [14,38-40]). In the absence of a recognized predefined taxonomy for DHTs, the DHTs used in the sources were charted iteratively by the type of technology [41] from the information provided about the intervention. Over time, DHT categories emerged and were defined (Multimedia Appendix 4). Discrete BCTs were identified from the intervention detail provided using the BCTTv1 [22] (Multimedia Appendix 5 [42]). A pragmatic decision was made that the single reviewer who had completed the BCTTv1 web-based training package [36] would code the interventions to the BCTTv1. Any areas of uncertainty were discussed in detail among the review team.

### Synthesis of Results

In accordance with the aims of a scoping review, formal assessments of methodological quality were not completed [30,31]. Findings were synthesized using descriptive statistics facilitated by SPSS Statistics 28.0.0.0 (IBM Corp) and Microsoft Excel (version 2208; Microsoft Corporation) and presented in text, table, and chart formats. The characteristics of the included sources, specifically participant numbers, age, and time since stroke, and intervention details, were summarized to provide contextual information for the review. Time since stroke was based on a published timeline framework [43], which describes the following phases: acute (1-7 days), early subacute (7 days to 3 months), late subacute (3-6 months), and chronic (greater than 6 months).

The behavior change theories, models, or frameworks underpinning the DHT-based interventions and sources where interventions had already been coded to the BCTTv1 were summarized. The use of individual BCTs, as coded by reviewers from intervention descriptions, was briefly summarized; however, the main focus of the BCT synthesis was completed by grouping the BCTs into the 16 BCTTv1 clusters, in order to provide an overview of their use across the sources and allow comparison with other reviews [44,45]. A cluster was only identified once per source, irrespective of the number of individual BCTs within that cluster. Relationships between BCTTv1 clusters and between DHT type and BCTTv1 clusters were descriptively explored. A relationship map was used to visually represent the strength of the connections between the BCTTv1 clusters, with a thicker line indicating that variables were more frequently reported together. No inferential statistical analysis was used.

## Results

### Selection of Sources of Evidence

**Figure 1:**
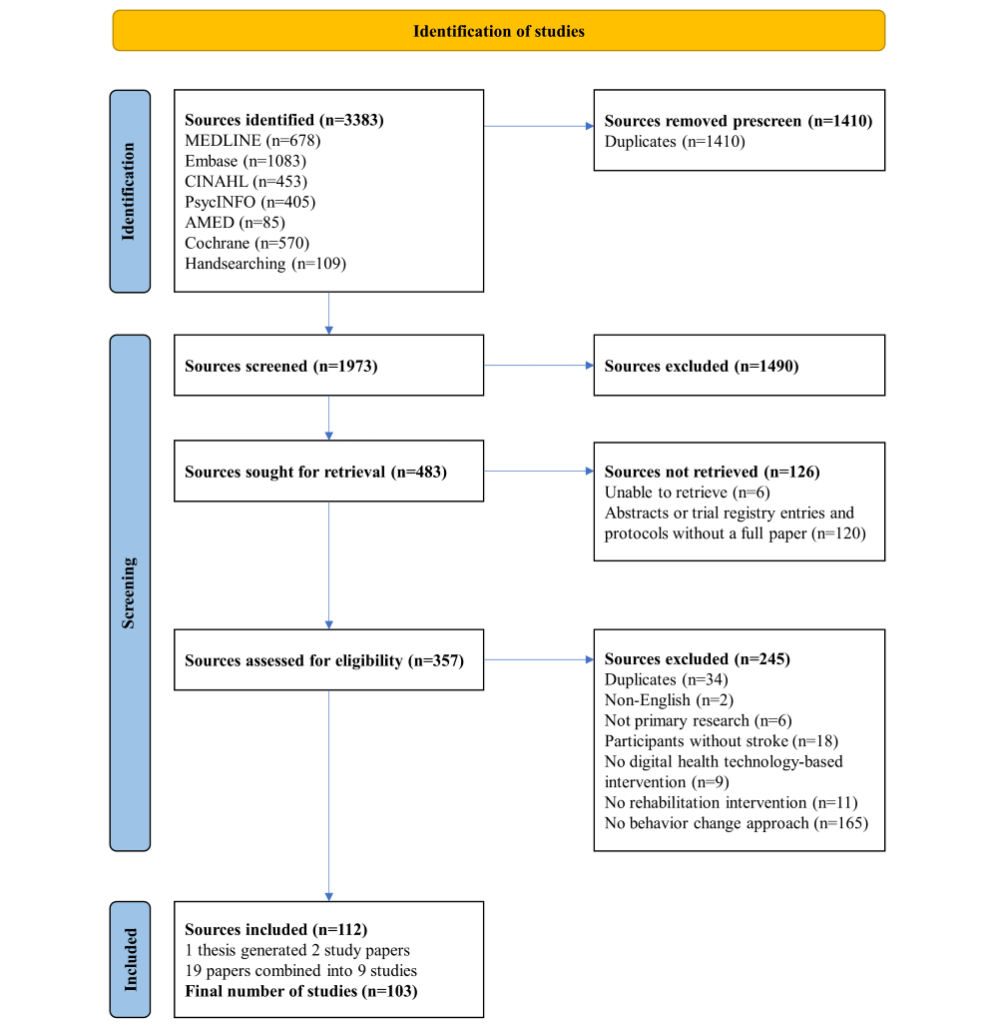
PRISMA (Preferred Reporting of Systematic Reviews and Meta-Analyses) flow diagram of the source selection process.

From a total of 1973 sources screened, 357 full-text sources were assessed for eligibility, then after grouping sources that referred to a single study, 103 (5%) distinct sources were included in the review [46-148] (Figure 1). Of the 245 sources excluded at full-text screening, 165 (67%) were excluded due to a lack of a behavior change approach.

### Characteristics of Sources of Evidence

#### General

**Figure 2:**
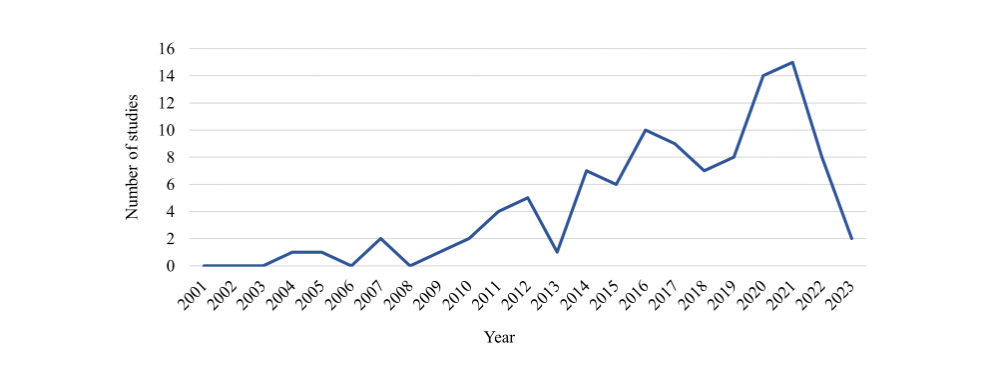
Number of studies by publication year (January 1, 2001, to March 21, 2023).

All sources of evidence were studies and will be referred to as such hereinafter. The number of studies in this field has rapidly increased over time (Figure 2), from a single study in 2004 to 8 in 2022, with a peak of 15 in 2021. The majority (86/103, 83%) [47-51,53-56,58,59,61,63-68,71-86,89-95,97-105,107, 109,111,112,114,115,117-126,128-136,138-148] were published in the past 10 years. Most studies took place in North America (41/103, 40%) [46-49,52,55,56,60,64-67,69,70,72,74,76-78,80, 85-88,92,93,97,99,101,108-110,126-129,137,138,141,142,145] and Europe (35/103, 34%) [51,53,54,57,58,62,63,68,71,79, 81-84,89,111,113-125,132,136,140,143,146,147], with the remainder in Asia (16/103, 16%) [50,59,61,91,94,95,98,100, 102-104,107,135,139,144,148], Australasia (9/103, 9%) [75,96,105,106,112,130,131,133,134], Africa (1/103, 1%) [90], and a single multicontinental study (1/103, 1%) [73]. Almost half (45/103, 44%) the studies are reported as feasibility or pilot studies [49,56,58,64,66,68,69,72-74,76,77,79,82-84,89,90,92,93, 95,97,100-104,106,108,114,116,117,119,122,124-126,131,134,136,138, 139,141,143,147]. Other study designs included randomized controlled trials (20/103, 19%) [50,51,60,61,65,75,80,85,86, 91,107,109,112,128-130,137,144,146,148], single session investigations (19/103, 18%) [47,52,57,59,71,78,87,88, 98,110,115,118,120,123,127,132,133,135,142], nonrandomized experimental designs (13/103, 13%) [53-55,62,63,67,81,94, 96,99,105, 113,145], case studies (4/103, 4%) [46,48,70,140], and realist evaluations (2/103, 2%) [111,121].

#### Participants

There were a total of 2825 participants in the 103 included studies. Studies tended to be small, with a median of 16 participants and a range of 1-188. Only half (55/103, 53%) the studies [46-48,50,56,57,59,61,64,67,69-72,78,79,82,87,88,92, 93,95-99,101,102,105,106,108,111-121,123-127,134, 138-140,142,143,145, 147] reported the minimum and maximum age of participants, which ranged from 17 to 99 years. Over three-quarters (83/103, 81%; 2508 participants) of studies reported the time since the onset of stroke. Of these 83 studies, 1 (1%; 48 participants) study [91] was conducted in the acute phase, 14 (17%; 504 participants) studies [60,61,68,74,79,92, 100,102,109,114,133,144,146,148] were conducted in the early subacute phase, 11 (13%; 316 participants) studies [59,65,66,72, 75,76, 81,104,107,121,134] were conducted in the late subacute phase, and 57 (69%; 1640 participants) studies [46,48,49,51, 53,54,57,63,64,67,69,70,73,78,80,82,84,85,88,89,93-99, 101,103,105,106,108,111-113,117-120,122-125,127-131,136-142, 145,147] were conducted in the chronic phase [43].

#### Study Intervention

An overview of study intervention characteristics is provided (Table 1). Interventions were focused on upper limb rehabilitation in almost two-thirds (65/103, 63%) of the studies [46-49,51,54-59,62-65,68,71,72,74,75,77-81,85-88,92,95, 96,99,101-103,105-108,110,112,113,116-118,121, 123-125,127,128,132,133,135-137,139-142,144-147]. Nearly all interventions (96/103, 93%) [46-80,84-94,96-117,119-121, 124-148] were delivered to individual participants, with over half (62/103, 60%) [46-50,53-58,60,61,64-70,72,74-77, 79,80,82-86,89,90,93,94,96,97,99,101,105,111, 112,116,117,119-122,126,129-131,134,136,138, 139,141,143-145,147] delivered fully or partly in the participant’s homes. Two-thirds (70/103, 68%) of studies [46-50,52-54,57,60,62,63,65-74,76-84,86-93,98,100,102, 104,108,109,112-115,117,118,120,122-125,129-131, 135-138, 140-142,144-146,148] included partial or full supervision of the intervention, with this predominately being provided face-to-face (48/70, 69%) [46,47,52,57,60,62,63,67,68,71,73, 78,81-84,86-89,91,92,98,100,102,104,108,109,112-115,117, 118,120,122-125,135-137,140,142,144-146,148]. Interventions lasted between a single session and 26 weeks.

Of the 103 studies, over half (n=57, 55%) of the studies [46,47,51-54,57,61,63,67,68,70,71,73,75-78,81,84-86,88-91,93, 95,96,98,100,102-104,106,109,112,114,115,123-126,129,130,132,133,135-138, 140,143-147] included 1 type of DHT, 30 (29%) studies [48,49,55,56,58-60,62,64,69,83,92,94,97,99,101,105,107,108,110, 111,113,116,118,121,122,127,128, 139,142] included 2 types, and 16 (16%) studies [50,65,66,72,74,79, 80,82,87,117,119,120, 131,134,141,148] included 3 types. VR was the most frequently used DHT (58/103, 56%) [46-49,51-53,57,59,62,63, 65,66,69,71,72,74,77,78, 80,81,84-89,92,95,96,98,102-104,106, 112,113,115,117-120,123-128,132,135-137,140, 142,143,146-148] followed by apps (31/103, 30%) [50,55,56,58,61,64-66,72,74,75,79,82,83,94,97,99,101,105,108,111, 114,116,119-122,131,134,139,141]. Further information on intervention characteristics with detail on associated citations is available (Multimedia Appendix 6 [46-148]).

**Table 1:** Intervention characteristics.

Intervention characteristics and details of characteristics			Studies (n=103), n (%)
**Intervention focus**			
	Upper limb	65 (63)	
	Multifocus	13 (13)	
	Physical activity	10 (10)	
	Mobility	5 (5)	
	Activities of daily living	4 (4)	
	Lower limb	4 (4)	
	Balance	1 (1)	
	Speech	1 (1)	
**Individual or group intervention**			
	Individual	96 (93)	
	Group	2 (2)	
	Combination	4 (4)	
	Not reported	1 (1)	
**Intervention location**			
	Home	48 (47)	
	Home and another setting	14 (14)	
	Health care setting	19 (18)	
	Research setting	12 (12)	
	Not reported	10 (10)	
**Supervision**			
	Both supervised and unsupervised	38 (37)	
	Supervised	32 (31)	
	Unsupervised	23 (22)	
	Not reported	10 (10)	
**Supervision contact**			
	Face-to-face	48 (47)	
	Remote	12 (12)	
	Combination (F2F^a^ and remote)	10 (10)	
	Not supervised or unreported	33 (32)	
**Number of DHT^b^ types**			
	1	57 (55)	
	2	30 (29)	
	3	16 (16)	
**DHT type**			
	VR^c^	58 (56)	
	App	31 (30)	
	Sensor	17 (17)	
	Activity monitor	16 (16)	
	Audio-video platform	15 (15)	
	Robotics	13 (13)	
	Messaging platform	11 (11)	
	Other	4 (4)	

^a^F2F: face-to-face.

^b^DHT: digital health technology.

^c^VR: virtual reality.

### Synthesis of Results

#### Behavior Change Theories, Models, and Frameworks

Most studies (93/103, 90%) [46-49,51-62,64-73,75-89,91-93, 96-106,108-115,117-137,139, 140,142-148] endeavored to link the intervention to behavior change; however, in the majority of these studies (75/93, 81%) [46,51-56,58-62,64-69,71-73,75, 77-89,91-93,96,97,99-101,103-106,108,110,112,114,115,117-120, 123,124,127,128,131-137,139,140,142-144,146-148], this explanation was centered on the reporting of the techniques perceived to change behaviors without direct reference to use of the BCTTv1 or on the reporting of a component of the intervention or the whole of the intervention as motivating. These explanations lack detail on how or why this influences behavior change. Examples of this included “the app also provided performance feedback, allowing the user to compare their current performance against their score from the previous session” (Bhattacharjya et al [56]) and “games motivate patients to engage in enjoyable play behavior” (Cramer et al [66]). A limited number of studies (18/103, 17%) [47-49,57,70,76,98, 102,109,111,113,121,122,125,126,129,130,145] articulated 1 or more theories, models, or frameworks of behavior change. While it is acknowledged that the BCTTv1 is a taxonomy framework rather than a theoretical framework, for the purpose of this review, it has been included as a framework for behavior change. A total of 13 different theories, models, or frameworks were identified within these 18 studies, with social cognitive theory being the most frequently reported (6/18, 33%) [76,109,111,121,129,130], followed by the behavior change technique taxonomy (4/18, 22%) [48,49, 122,129], game design theory (3/18, 17%) [47,57,125], operant conditioning (3/18, 17%) [47,98,121], and self-determination theory (3/18, 17%) [48,49,126]. Further information on behavior change theories, models, and frameworks, with details on associated citations, is available (Multimedia Appendix 7 [47-49,57,70,76,98,102, 109,111,113,121,122,125,126,129,130,145]).

#### Behavior Change Techniques

Despite 4 studies acknowledging the BCTTv1, explicit BCTTv1 codes were only reported in 2 studies (2/103, 2%) [48,122]. However, a third study (1/103, 1%) mapped the techniques used to change behavior directly to the transtheoretical model [145]. There was a median of 3 (range 1-14) individual BCTs coded per study, with a total of 383 BCTs across the 103 studies. The most frequently identified individual BCTs were feedback on behavior and nonspecific reward (Multimedia Appendix 8).

There was also a median of 3 (range 1-8) BCTTv1 clusters per study, with a total of 288 clusters coded across the 103 studies. The most frequently used of the 16 possible clusters were feedback and monitoring (88/103, 85%) [46-60,62-69, 71-74,76,78-80,82-92,94-106,108-113,116,117,119-129,134-146,148], reward and threat (56/103, 54%) [46-49,51-53,55-57,62,65,69, 71,72,74,77,80,81,85,86,88,89,91,92,95,96,98,102,103,106-108,112,113,115, 117-119,121-125,128,132,134-137,140,142,143,146-148], goals and planning (33/103, 32%) [49,58,60,65-68,70,72,74,76,79,80, 82,83,90,91,93,94,97,100,109,111,112,121,122,126,129,130,134,138, 141,145], and shaping knowledge (33/103, 32%) [46,48,50, 53-56,58,60,61,64-72,74,75,86,94,97,101-103, 108,111,113,114,120,129-131,139-141]. Other BCTTv1 clusters used were social support (24/103, 23%) [48,49,58,60,64,67,70,72,73,79,80,82,84,90,93, 101,108,117,119,129-131,134,141], comparison of behavior (23/103, 22%) [46,50,53,54,60,61,64-66,74,75,81,86,101, 104,111,114,118,122,123,125,131,139], associations (16/103, 15%) [58,60,65,66,68,75,80,83,87,90,110,120,131,133,139,144], repetition and substitution (6/103, 6%) [60,82,109, 122,129,130], scheduled consequences (3/103, 3%) [47, 80,88], natural consequences (2/103, 2%) [129,138], comparison of outcomes (2/103, 2%) [47,133], antecedents (1/103, 1%) [60], and self-belief (1/103, 1%) [70]. The clusters of regulation, identity, and covert learning were not identified. Within the context of the review, it was noted that the reward and threat cluster only included reward-based BCTs. A tabulated summary and graphical representation of the BCTTv1 clusters is available (Multimedia Appendix 9 [46-148]).

**Figure 3:**
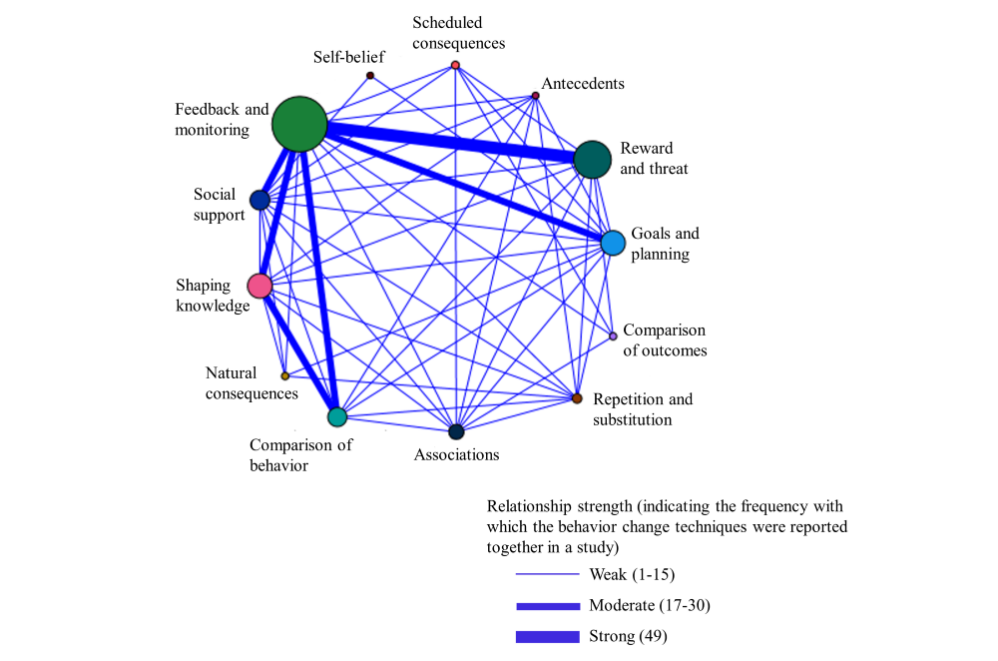
Relationships between behavior change technique taxonomy clusters.

The exploration of clusters that were reported together in an intervention (Figure 3) identified the strongest relationship between the clusters of feedback and monitoring and reward and threat. Clear links were also identified between feedback and monitoring and 4 other clusters: goals and planning, shaping knowledge, social support, and comparison of behavior, and between the shaping knowledge and comparison of behavior clusters.

#### Behavior Change Techniques and Digital Health Technology

**Figure 4:**
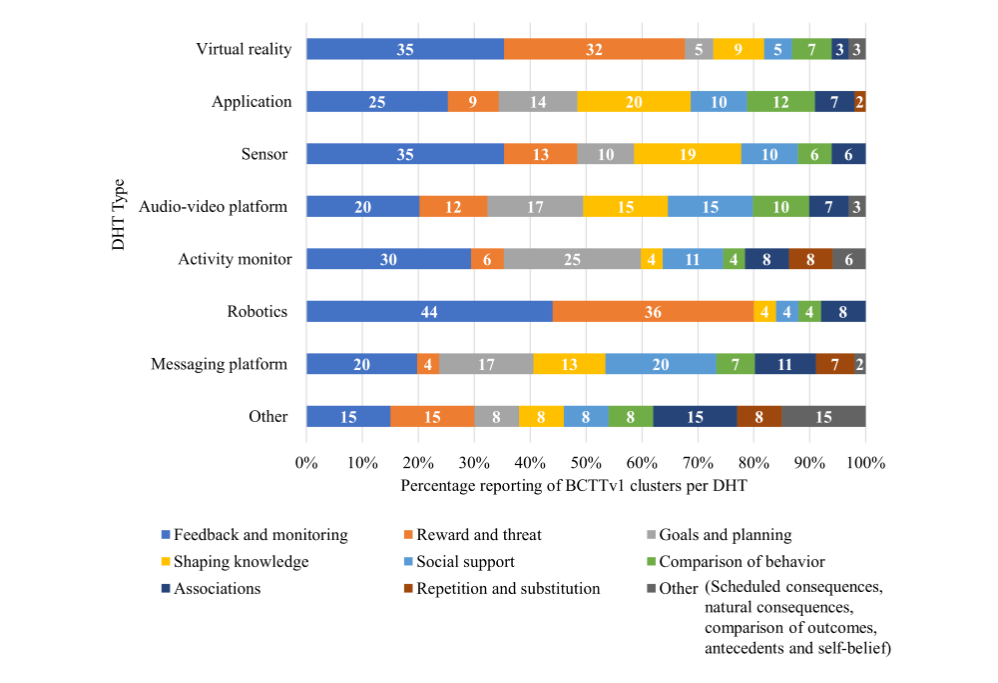
Percentage of behavior change technique taxonomy clusters use across the different types of digital health technology. BCTTv1: behavior change technique taxonomy version 1; DHT: digital health technology.

The feedback and monitoring cluster was reported most frequently for all types of DHT (Figure 4), with the greatest proportion of this cluster in robotics (11/25, 44%) [59,62,87,92,110,113,117,127, 128,142,148], VR (52/148, 35%) [46-49,51-53,57,59,62,63,65,66,69,71,72,74,78,80,84-89,92, 95,96,98,102-104,106,112,113,117,119,120,123- 135-137,140, 142,143,146,148], and sensors (17/48, 35%) [50,55,56,87,94,99, 101,105,108,110, 111,116,119-121,134,141]. Robotics and VR also often used the reward and threat cluster (9/25, 36% [62,92, 107,113,117,118,128,142,148] and 48/148, 32% [46-49,51-53, 57,62,65,69,71,72,74,77,80,81,85,86,88,89,92,95,96,98,102,103,106, 112,113,115,117-119,123-125,128,132,135-137,140,142,143,146-148], respectively), while the goals and planning cluster was a dominant second cluster in activity monitors (13/53, 25%) [67,68,76,79, 80,82,91,100, 109,122,129,138,145].

## Discussion

### Summary of Evidence

This scoping review provides a comprehensive overview of approaches used to support changes in behavior in DHT-based physical stroke rehabilitation interventions. Research in this field is in its infancy, with the predominance of studies in this review being described as pilot or feasibility studies with limited participants.

Despite using comprehensive behavior change search terms, only a limited number (103/1973, 6%) of screened sources were included. Over two-thirds of full-text sources were excluded as they did not describe or refer to any behavior change theories, models, or frameworks or BCTs, suggesting that in general, explicit behavior change approaches are not reported as being integral to DHT-based physical stroke rehabilitation.

Only 18 (17%) of the 103 included studies articulated a theory, model, or framework to underpin the intervention, which aimed to change behavior, despite widely published recommendations about the importance of overt use of theory when developing, evaluating, and reporting interventions [27,29], including those related to behavior change [28]. The proportion of studies articulating a behavior change theory, model, or framework in this work is significantly lower than review findings in non-rehabilitation DHT-based interventions that have sought to influence specific behaviors such as physical activity or weight control [24,44]. These reviews have identified up to two-thirds of sources reporting a theory, model, or framework. However, our findings mirror the relative absence of behavior change theories, models, and frameworks in rehabilitation interventions more generally, irrespective of whether they use digital technology [149] or not [45], and it is widely recognized that the complex nature of rehabilitation often results in the essential characteristics of interventions being poorly defined [150]. Consistent with our findings in these other reviews, a variety of theories, models, and frameworks were found to underpin interventions, with social cognitive theory being the most frequently reported [24,44,45,149]. The explicit description of BCTs using the BCTTv1 within DHT-based physical stroke rehabilitation interventions is also poorly reported (2%), despite a significant proportion of the sources being dated after the publication of the BCTTv1 in 2013 [22]. This lack of acknowledgment of behavior change approaches impedes the accumulation of knowledge within this field.

It is important that both the underpinning theory and BCTs are reported so the mechanisms by which the BCTs elicit change can be better understood [21]. The general assumption that the motivational and captivating aspects of DHTs will promote prolonged and repeated engagement with rehabilitative activities, in particular in those DHTs that incorporate game design [151], risks suboptimal outcomes for patients and wasted investment of time and money if the mechanisms by which the DHT elicits change are not considered.

When exploring which BCT clusters featured within the reviewed DHT-based interventions, the findings relating to the commonly used clusters of feedback and monitoring, goals and planning, and shaping knowledge are consistent with findings from DHT-based interventions to change a specific behavior [44] and non-DHT–based rehabilitation [45]. However, a novel finding in our review was the frequent identification of the reward and threat cluster, although it was noted that all techniques related to reward and none to threat. A large number of studies in this review used VR technology, which frequently incorporates gamified tasks or gameplay. Reward is an integral part of game design theory alongside feedback [152], and so it is perhaps unsurprising that the feedback and monitoring, and reward and threat clusters dominated and an association between these 2 clusters was seen.

### Limitations

Rehabilitation is a process that comprises multiple behaviors and so exploring approaches to change behavior within this context was complicated. There were challenges in searching and screening sources for inclusion as few studies explicitly reported approaches to change behavior, and there is a similarity in the vocabulary used within behavior change and other theoretical approaches (eg, “feedback,” which is used within motor learning). Similarly, only a very small proportion of studies explicitly reported BCTs within interventions. The lack of clear reporting of behavior change introduces the risk that sources may be omitted during both the searching and screening process highlighting the difficulty of comprehensively reviewing this field of work. An inclusive approach to screening reduced the risk of erroneously excluding sources, but it is perhaps inevitable that the sources included reflect those studies that have reported a behavior change approach rather than all studies that have used one.

This lack of clear BCT reporting also posed challenges for intervention coding. The use of the BCTTv1 aimed to ensure the review used a generalizable nomenclature to describe BCTs, and the 1 reviewer who had completed BCTTv1 training coded all the interventions. It is acknowledged that decisions made in the application of the BCTTv1 within the context of the review will have introduced some subjectivity in intervention coding, which will ultimately influence the review findings. Although the coding process could have been made more robust by having a second reviewer trained in the BCTTv1 also code the interventions, regular and extensive discussions between all members of the review team took place with the aim of ensuring consistency with the coding process. Clear documentation as to how the BCTTv1 was used within this review (Multimedia Appendix 5) supports transparency as to the decisions made and the reproducibility of the review.

The absence of a recognized predefined taxonomy for DHTs posed a challenge when categorizing the DHT interventions, acknowledging that the distinction between the categories used to present the results is open to interpretation. A description of how the reviewers interpreted these categories is provided (Multimedia Appendix 4).

### Implications for Research

Future studies aimed at developing and evaluating DHT-based rehabilitation interventions, including those relating to physical stroke rehabilitation, need to ensure there is explicit recognition and reporting of the specific approaches used to change behavior, articulating both the theory on which the intervention is based and how the intervention plans to deliver the change in behavior using universally recognized terminology. This should be reported as part of a program theory and potential mechanisms of action, which are key parts of developing and evaluating complex interventions [29]. This detailed reporting would further support an understanding of how changes in behavior could be best enabled by DHT-based rehabilitation interventions and how this contributes to changes in patient outcomes. It would also enable further evaluation of the optimal behavioral components of interventions, enabling patients to use and clinicians to deliver the most effective DHT-based rehabilitative interventions. More generally, as the use of DHTs expands, there is an urgent need for some form of taxonomy to categorize and clearly define the different types of DHTs to facilitate consistent reporting, replication, and comparison of DHT-based interventions.

### Conclusion

This novel and original review is the first to explore if and how approaches to change behavior are incorporated within DHT-based physical stroke rehabilitation. It demonstrates that a minority of studies report using approaches to change behavior within this context, despite these changes in behavior being vital to meet the demands of rehabilitative activities. Those who do report behavior change often lack the underpinning detail as to how the DHT-based intervention will facilitate these changes. In order for DHT-based interventions to realize their potential within rehabilitation and their impact on patient outcomes, approaches to change behavior must be embedded in the intervention and appropriately reported.

## Appendix

Multimedia Appendix 1 Preferred Reporting of Systematic Reviews and Meta-Analyses Extension for Scoping Reviews (PRISMA-ScR) checklist.

Multimedia Appendix 2 Inclusion and exclusion criteria.

Multimedia Appendix 3 Full search strategy as used in Medline.

Multimedia Appendix 4 Data charting tool.

Multimedia Appendix 5 Review-specific behavior change technique taxonomy coding decisions.

Multimedia Appendix 6 Intervention characteristics (with associated references).

Multimedia Appendix 7 Behavior change theories, models, and frameworks reported (with associated references).

Multimedia Appendix 8 Individual behavior change techniques coded.

Multimedia Appendix 9 Behavior change technique taxonomy clusters identified (with associated references).

## Abbreviations

BCT: behavior change technique
BCTTv1: behavior change technique taxonomy version 1
DHT: digital health technology
PRISMA-ScR: Preferred Reporting of Systematic Reviews and Meta-Analyses Extension for Scoping Reviews
TIDieR: Template for Intervention Description and Replication
VR: virtual reality

## Data Availability

All data supporting this study are openly available from the University of Central Lancashire repository [153].
