# Supplementary material for: Behavior Change Approaches in Digital Technology–Based Physical Rehabilitation Interventions Following Stroke: Scoping Review
Source: J Med Internet Res. 2024 Apr 24;26:e48725. doi: 10.2196/48725 (PMC11079774; doi:10.2196/48725)
Supplement: Multimedia Appendix 2 [file jmir_v26i1e48725_app2.pdf]

Multimedia Appendix 2. Inclusion and exclusion criteria

Full text exclusions will report exclusion reason based on highest category (1 highest, 7 lowest).

Category	Inclusion	Exclusion
**1. Date**	2001-present	Pre 2001
**2. Language**	English	Non-English
**3. Study type**	Completed primary research study (any study design) including digital health technology (DHT) development studies during which the intervention has been trialled	Protocols and trial registry entries if a completed study is not found (excluded at full text searching)<br>Abstract only without a full publication ((excluded at full text searching)<br>Review or commentary<br>DHT development papers prior to stage of participant trial
**4. Participants involved in Intervention**	Adults<br>Diagnosis of stroke with a physical impairment in function as defined by the International Classification of Functioning, Disability and Health of<br>a) Sensory functions and pain<br>b) Neuromusculoskeletal and movement related functions (including swallow)<br>c) Voice and speech<br>Mixed conditions and adult/child participant groups where adult stroke participant data can be separated	Non-human<br>Entire cohort under 18 years<br>No diagnosis of stroke<br>Diagnosis of stroke with only cognitive impairment as defined by the International Classification of Functioning, Disability and Health of mental function including cognition, language, visuospatial attention<br>Mixed conditions and adult/child participant groups where adult stroke participant data cannot be separated

**5. Interventions DHT**	Specific DHT identified by use of hardware and/or software	No specific DHT identified<br>Intervention only uses telephone calls or email as a means of communication<br>Use of electrical stimulation alone<br>Use of videos via video player
**6. Interventions Physical Rehabilitation**	Active physical rehabilitation interventions to maintain or reduce disability, optimize function, and facilitate participation in meaningful life tasks (includes risk management and health promotion if physical activity is included)	No rehabilitation intervention<br>Intervention for assessment only<br>Interventions which are fully assistive<br>Rehabilitation of mental (cognitive) function<br>Interventions to address medication adherence or diet
**7. Interventions Behaviour Change**	An explicit mention of a behaviour change theory, framework, model, or behaviour change technique (BCT) within the DHT intervention description<br>Note- At title and abstract screening stage all articles with a potential for behaviour change will be included for full text screening and at full text screening the behaviour change technique taxonomy v1 (BCTTv1) coding principles will be applied	No explicit behaviour change approach within the DHT intervention<br>An intervention which includes BCTs and uses a DHT, but they are used completely separately<br>A potential BCT such as feedback is explicitly referenced to another theory e.g., motor learning theory without consideration of behaviour change<br>Intervention not described in sufficient detail for BCTs to be identified and coded
