# Supplementary material for: Behavior Change Approaches in Digital Technology–Based Physical Rehabilitation Interventions Following Stroke: Scoping Review
Source: J Med Internet Res. 2024 Apr 24;26:e48725. doi: 10.2196/48725 (PMC11079774; doi:10.2196/48725)
Supplement: Multimedia Appendix 3 [file jmir_v26i1e48725_app3.pdf]

Multimedia Appendix 3. Full search strategy as used in Medline

1	Behavior Therapy/ or Health Behavior/
2	(behavio* change or behavio* modification or behavio* outcome or behavio* strategy or behavio* facilitation or chang* behavio* or effect behavio* or influence behavio* or impact behavio* or health behavio*).ti,ab,kw.
3	(behavio* adj4 (model* or theor* or framework or taxonomy)).ti,ab,kw.
4	(affect infusion model or ASE-model or attitude social influence self-efficacy model or attribution theory or belief system theory or biopsychosocial model or change theory or classical conditioning or COM-B or control theory or diffusion of innovation theory or diffusion of innovations theory or disconnected values model or dual process theory or behavio* dynamics or dynamic systems theory or ecological model or ecological momentary assessment or elaboration likelihood model or empowerment theory or expected utility theory or expectancy value theory or information processing model or extended parallel process model or family systems theory or FRAMES Feedback Responsibility Advice Menu of options Empathy Self-efficacy or habit theory or health action process approach or health belief model or health communication theory or integrated change model or I-change model or integrat* model or interactionist model or information processing model or attitude change or intervention mapping or intrapersonal theory or interpersonal theory or knowledge attitude behavio*).ti,ab,kw.
5	(model of human occupation or multicomponent stage model or narrative persuasion or needs opportunities abilities model or network theory or norm activation model or normative conduct or operant conditioning or precaution adoption process model or pressure system model or reciprocal determinism or reciprocal causality or reflective impulsive model or regulatory fit theory or risks as feelings model or salutogenic model or self-determination theory or self-perception theory or six staged model or social* theory or social* model or social consensus or stage change model or technology acceptance model or temporal construal theory or theoretical domains framework or theory of planned behavio* or theory of reasoned action or theory of triadic influence or transtheoretical model or transtheoretical theory or utility theory or value belief norm).ti,ab,kw.
6	(motivation* adj4 (theory or model)).ti,ab,kw.
7	(BCT or BCI or behavio* therapy or behavio* intervention).ti,ab,kw.
8	(action planning or behavio* contract or (goal adj4 (behavio* or review or outcome or setting))).ti,ab,kw.
9	(behavio* adj4 (feedback or monitoring)).ti,ab,kw.
10	(behavio* adj6 (barrier or problem-solving or biofeedback or instruction or antecedent or re-attribution or experiment* or demonstrat* or compar* or consequence or cost or commitment or avoidance or anticipated regret or distraction or reward or punishment or incentive or motivation or prompt or cue or reminder or persuasion or approval or stimulus or exposure or associative learning or restructuring or adding objects or target or practice or substitution or overcorrection or graded task or credible source or role model or framing or reframing or incompatible beliefs or success or support or reduce negative emotions or conserving mental resources or body changes or mental rehearsal or habit)).ti,ab,kw.
11	(behavio* adj20 (self-management or self-care or self-belief or self-efficacy or self-regulation or self-identity or self-talk or coaching)).ti,ab,kw.
12	((participation or adherence or compliance or engagement) adj8 (rehabilitation or intervention or therapy or treatment or exercise)).ti,ab,kw.
13	or/1-12
14	Telemedicine/ or Telerehabilitation/ or Remote Consultation/ or Digital Technology/ or User-Computer Interface/ or Mobile Applications/ or Therapy, Computer Assisted/
15	(digital technolog* or health technolog* or e-health or m-health or ehealth or mhealth or mobile health or digital health or digital healthcare or telemedicine or tele-medicine or telehealth or tele-health or telecare or tele-care or telemanagement or tele-management or telerehab* or tele-rehab* or teleconsultation or tele-consultation or computer-based based therapy or computer-based medicine or computer assisted therapy or computer assisted medicine or remote consultation or remote monito* or remote care or remote treatment or remote therap* or health informatics or

	medical informatics or biomedical technolog* or patient portal or virtual rehabilitation or virtual consultation or virtual therap* or virtual treatment or virtual care or digital monitor*).ti,ab,kw.
16	((digital device or smart-phone or iPod or iPad or android or apple or blackberry or tablet or smart-watch or computer or PC or microcomputer or Fitbit or pedometer or X-Box or Kinect or Wii or wireless or Nintendo or PlayStation or games console) adj10 (rehabilitation or intervention or therapy or treatment or exercise)).ti,ab,kw.
17	(personal digital assistant or activity tracker or remote sensing technolog* or wearable sensor* or wearable technolog* or interactive technolog* or VR or augmented reality or mixed reality or leap motion or robotics or artificial intelligence).ti,ab,kw.
18	((digital interaction or app or application or digital medi* or internet or web-based or online or video or YouTube or skype or zoom or e-mail or electronic mail or text messag* or SMS or MMS or Wi-Fi) adj10 (rehabilitation or intervention or therapy or treatment or exercise)).ti,ab,kw.
19	(serious gam* or applied gam* or health gam* or exergam* or rehabilitation gam*).ti,ab,kw.
20	or/14-19
21	Physical Therapy Modalities/ or Physical Therapy Specialty/ or "Physical and Rehabilitation Medicine"/
22	Neurological Rehabilitation/ or Rehabilitation/ or Stroke Rehabilitation/ or Cardiac Rehabilitation/
23	Exercise Therapy/ or Recreation Therapy/ or Early Ambulation/ or Exercise/
24	"Recovery of Function"/ or Occupational Therapy/ or Allied Health Occupations/ or Therapeutics/ or Physical Therapist Assistants/ or Allied Health Personnel/ or "Activities of Daily Living"/
25	(Physiotherap* or physical therap* or rehabilitation or rehabilitative or neurorehabilitation or mobili#ation or (activities adj2 daily living) or functional activit* or functional training or exercise or ambulation or mobility or functional assessment or recreational therap* or occupational therap* or allied health worker* or allied health professional*).ti,ab,kw.
26	or/21-25
27	cerebrovascular disorders/ or exp basal ganglia cerebrovascular disease/ or brain ischemia/ or exp brain infarction/ or ischemic attack, transient/ or vertebrobasilar insufficiency/ or exp carotid artery diseases/ or cerebral small vessel diseases/ or cerebral amyloid angiopathy, familial/ or stroke, lacunar/ or cerebrovascular trauma/ or vertebral artery dissection/ or intracranial arterial diseases/ or cerebral arterial diseases/ or cerebral amyloid angiopathy/ or infarction, anterior cerebral artery/ or infarction, middle cerebral artery/ or infarction, posterior cerebral artery/ or moyamoya disease/ or intracranial aneurysm/ or intracranial arteriosclerosis/ or exp intracranial arteriovenous malformations/ or exp "intracranial embolism and thrombosis"/ or intracranial hemorrhages/ or exp cerebral hemorrhage/ or intracranial hemorrhage, hypertensive/ or exp subarachnoid hemorrhage/ or stroke/ or vasospasm, intracranial/
28	Brain Injury, Chronic/
29	Stroke Rehabilitation/
30	(stroke* or cva* or poststroke or post-stroke or cerebrovasc* or (cerebr* adj3 vasc*)).tw.
31	((cerebr* or cerebell* or vertebrobasil* or brain) adj3 (isch?emi* or infarct* or thrombo* or emboli* or apoplex*)).tw.
32	((cerebr* or cerebell* or vertebrobasil* or subarachnoid* or arachnoid* or brain) adj3 (h?emorrhag* or h?ematom* or bleed*)).tw.
33	hemiplegia/ or exp paresis/ or exp gait disorders, neurologic/
34	(hempar* or hemipleg* or paresis or paretic or brain injur*).tw.
35	or/27-34
36	13 and 20 and 26 and 35
37	limit 36 to (english language and yr="2001 -Current")
