# Supplementary material for: Behavior Change Approaches in Digital Technology–Based Physical Rehabilitation Interventions Following Stroke: Scoping Review
Source: J Med Internet Res. 2024 Apr 24;26:e48725. doi: 10.2196/48725 (PMC11079774; doi:10.2196/48725)
Supplement: Multimedia Appendix 4 [file jmir_v26i1e48725_app4.pdf]

Multimedia Appendix 4. Data charting tool

Data charting		Notes	Pre-set options
**Study Details**			
	Author(s)		
	Title		
	Year of publication		
	Grouped country of origin	Multi-continental – intervention took place across two or more continents	North America<br>Europe<br>Asia<br>Africa<br>Australasia<br>South America<br>Multi-continental
**Methods**<br>If a study includes a single session followed by a further longer study period, the details from the longer study period will be recorded			
	Study design	Usability study will be included under feasibility/pilot	Case study<br>Feasibility/pilot<br>Randomised Controlled Trial<br>Mixed methods<br>Non-randomised /-controlled experimental<br>Non-experimental/observational<br>Single session exploration<br>Qualitative<br>Other
	Specify if other		
	Participant numbers	Use participant numbers for which the participant demographics are reported	
	Participant characteristics/ age/ range lowest		
	Participant characteristics/ age/ range highest		
	Participant characteristics/ time since stroke in months*	Conversion to months: years x 12, weeks ÷ 4.3, days x 0.0328767123	

**Intervention (Based on the TIDieR Checklist)**<br>All information will relate to the intervention only i.e., not methods of data collection or evaluation and will be charted from the introduction and methods section (not the discussion)<br>If a DHT is also being used for other functions outside of the defined scope of rehabilitation e.g., BP monitoring, medication adherence then only the rehabilitation aspect will be charted<br>If a study is described in distinct stages and only some of those stages involve DHT then only this part will be data charted			
**TIDieR item**			
Why	Link between intervention and behaviour change approaches		Yes<br>No
	Description of link	Summarised by reviewer	
	Reference to a specific behaviour change theory framework or model	As defined by the original author	Yes<br>No
	Specify theory framework or model		
	Focus / goal of the intervention		Upper limb<br>Lower limb<br>Mobility<br>Physical activity<br>Balance<br>Speech and language<br>Multi-focus<br>Activities of daily living<br>Other
	Specify if other		
What	Number of DHT types		One<br>Two<br>Three
	Type(s) of DHT intervention	**Virtual Reality (VR)** – an artificial environment created in 3D (immersive) or on a visual display (non-immersive) with which the user can interact in real time [38], incorporating any game, activity or exercise<br>**App –** a computer programme used for a specific	VR<br>App<br>Audio-video platform<br>Robotics<br>Messaging platform<br>Activity Monitor

		purpose which can downloaded onto a device or accessed via web browser [39] excluding VR<br>**Audio- video platform -** all synchronous, combined audio and visual communication platforms excluding audio telephone calls<br>**Robotics –** a programmed device (end- effector or exoskeletal) which assists the user through a specific motion whilst providing resistance to undesired movements if indicated [40]<br>**Messaging platform** –all asynchronous communication platforms excluding telephone answering machines<br>**Activity monitor** – a wearable electronic device which delivers feedback about physical activity [14]<br>**Sensor** – a movement sensors which is worn or attached to an object to collect information or provide feedback excluding those that provide physical activity feedback and those associated with a VR system	Sensor<br>Other
	Specify if other		
	Behaviour change techniques (BCTs) coded to BCTTv1	See Multimedia Appendix 5 Review-specific behaviour change technique taxonomy coding decisions	
	Number of BCTs coded		
	Number of BCT clusters coded		
	BCTs coded by authors and documented in the source		Yes<br>No
How	Supervision	Synchronous contact with the participant either face to face or remotely during the intervention excluding initial session set up<br>Provided through the DHT and /or provided to support the use of the DHT	Yes<br>No<br>Both<br>Not reported
	If supervised specify if face to face or remote		Face to face<br>Remote

			Both<br>Not reported
	Individual or group intervention	Group is 2+ participants with stroke	Individual<br>Group<br>Both<br>Not reported
Where	Location of the intervention	Community includes a gym or community centre<br>Health care setting includes hospitals (in and out-patients) and community clinics<br>Research setting includes laboratories and universities	Home<br>Community<br>Healthcare setting<br>Research setting<br>Home and healthcare<br>Home and research<br>Home and community<br>Home and other (other not specified)<br>Not reported<br>Other
	Specify if other		
When and how much	Duration in weeks	Length of time (longest duration if a range is given) over which intervention is provided in weeks with single exploratory sessions identified as such.<br>Conversion to months: weeks ÷ 4.3	

*Use mean or median (mean used as preference unless there is evidence that the data is markedly skewed)
