# Supplementary material for: Behavior Change Approaches in Digital Technology–Based Physical Rehabilitation Interventions Following Stroke: Scoping Review
Source: J Med Internet Res. 2024 Apr 24;26:e48725. doi: 10.2196/48725 (PMC11079774; doi:10.2196/48725)
Supplement: Multimedia Appendix 5 [file jmir_v26i1e48725_app5.pdf]

Multimedia Appendix 5. Review specific behaviour change technique taxonomy coding decisions

The coding of interventions using the behaviour change technique taxonomy (BCTTv1) was completed in line with the BCTTv1 coding principles, however in the context of rehabilitation interventions some pragmatic decisions had to be made to ensure the most valuable information was collected and presented. These decisions were extensively discussed by the scoping review team and the following information produced for transparency.

**General**	The behaviour was the engagement with the specific rehabilitation intervention and the behaviour outcome was the impact of doing the intervention.<br>Coding was based on the intervention description in the introduction or methods not information explored in the discussion.<br>A code was only given once for an intervention.
**Goals and planning**	If the goal setting was unclear whether it focused on the behaviour or the outcome, then it was coded to 1.3 "Goal-setting outcome".<br>Goals embedded in virtual reality activities were not coded as goals.
**Feedback and monitoring**	Sources appeared to use interchangeable language in the text about feedback and reward especially in relation to game scores. Game scores and visual changes within a game in response to success / failure were coded as both 2.2 "Feedback on behaviour" and 10.3 "Non-specific reward". Feedback and reward were also coded separately when indicated.<br>2.6 "Biofeedback" is feedback on a physiological or biochemical state, therefore feedback on movement parameters (e.g., range of movement) was not coded as biofeedback. However, feedback at a physiological level (e.g., EMG activity) was coded as such.<br>If a provider monitored behaviour or outcomes of behaviour and then remotely or independently adjusted the intervention in response to this monitoring without explicit feedback to the participant, then codes 2.1 "Monitoring of behaviour by others without feedback" or 2.5 "Monitoring of outcomes of behaviour by others without feedback" were used. If the intervention was adjusted alongside feedback with the participant, then 2.2 "Feedback on behaviour" or 2.7 "Feedback on outcomes of behaviour" were coded.<br>If a digital health technology (DHT) was monitoring and giving feedback this was not coded as 2.3 "Self-monitoring of behaviour", however a participant adding data to a DHT programme was coded as such.
**Social support**	Encouragement which was provided by the DHT was coded as 3.1 "Social support unspecified".
**Shaping knowledge**	All DHT-based rehabilitation interventions are likely to include some initial instruction about using the DHT or on the exercises/activities to be completed to ensure understanding and safety however, code 4.1 "Instruction on how to perform a behaviour" was not used in this context. If additional instruction was provided during the intervention this was coded.
**Comparison of behaviour**	Some DHT- based rehabilitation interventions may include initial demonstration on using the DHT or on the exercises/activities to be completed and this was not coded as 6.1 "Demonstration of the behaviour". If additional demonstration was provided during the intervention this was coded.
**Repetition and substitution**	Frequently rehabilitation is trying to gain repetitive practice. This repetitive practice is part of the behaviour of the intervention and was not classed as a BCT. Any practice or rehearsal completed alongside initial information and demonstration was not coded.<br>Additional professionally supervised (real time) practice (face to face or virtual) to support changes in independent rehabilitation behaviour was coded as 8.1 "Behavioural practice/rehearsal".<br>8.7 "Graded tasks" required a clear grading of activity towards a target behaviour. A mention of progression of exercise/ increasing the difficulty of exercise without explicit links to a behaviour was not coded as graded tasks.

**Comparison of outcomes**	Due to the nature of clinical research, interventions are delivered by therapists / researchers who in themselves would be considered a credible source. The code 9.1 “Credible source” was only used where there was evidence that additional verbal/non-verbal communication was given in favour or against a behaviour as part of the intervention.
**Reward and threat**	See detail under Monitoring and Feedback.<br>A DHT recording number of repetitions was not classed as a score and therefore not coded under reward.<br>Verbal or non-verbal rewards were coded as 10.4 “Social reward”.<br>10.4 “Social reward” was only coded when it was delivered by a person not the DHT. Any reward from the DHT was coded as 10.3 “Non-specific reward”.
**Antecedents**	Most DHT- based rehabilitation interventions will add objects, i.e., the DHT hardware, to the environment, and code 12.5 “Adding objects to the environment” was not used in this context. Similarly physical exercise equipment or paying for data / Wi-Fi was not coded within this cluster. The antecedent cluster was only coded if adaptive equipment was provided to support a change in behaviour.<br>Code 12.5 “Body changes” was not coded for interventions if the change (e.g., in strength or movement) was the outcome of the behaviour and not a way of facilitating the behaviour.
**Scheduled consequences**	14.8 “Rewarding alternative behaviour” was not used in the context of reducing the unwanted behaviour.<br>Shaping is often discussed as a component of constraint induced movement therapy (CIMT). In this context a final desired activity is gained in a step wise approach, with each step becoming closer to the desired outcome [42]. Shaping within the context of the BCTTv1 is associated with reward approximation i.e., Arranging a reward following any approximation to the behaviour, gradually rewarding only performance closer to the wanted behaviour. Shaping was only coded as 14.4 “Reward approximation” if there was a clear link between progression and reward.
