# Supplementary material for: Behavior Change Approaches in Digital Technology–Based Physical Rehabilitation Interventions Following Stroke: Scoping Review
Source: J Med Internet Res. 2024 Apr 24;26:e48725. doi: 10.2196/48725 (PMC11079774; doi:10.2196/48725)
Supplement: Multimedia Appendix 6 [file jmir_v26i1e48725_app6.pdf]

Multimedia Appendix 6. Intervention characteristics (with associated references)

	Intervention Data	Studies (n=103), n (%)	Citations (as referenced in main manuscript)
**Intervention Focus**	Upper limb	65 (63%)	[46-49,51,54-59,62-65,68,71,72,74,75,77-81,85-88,92,95,96,99,101-103,105-108,110, 112,113,116-118,121,123-125,127,128,132,133,135-137,139-142,144-147]
	Multi-focus	13 (13%)	[50,53,61,66,84,89,104,111,119,120,126,130,143]
	Physical activity	10 (10%)	[67,76,82,91,94,100,122,129,131,138]
	Mobility	5 (5%)	[60,70,73,109,115]
	Activities of daily living	4 (4%)	[83,90,93,97]
	Lower limb	4 (4%)	[52,69,134,148]
	Balance	1 (%)	[98]
	Speech	1 (%)	[114]
**Individual or Group Intervention**	Individual	96 (93%)	[46-80,84-94,96-117,119-121,124-148]
	Group	2 (2%)	[81,123]
	Combination	4 (4%)	[82,83,118,122]
	Not reported	1 (1%)	[95]
**Intervention Location**	Home	48 (47%)	47-50,53,55-58,60,61,64-66,69,70,72,75-77,84,85,90,93,94,96,97,99,101,105,111,116, 117,119-121,126,129,130,134,136,138,139,141,143-145,147]
	Home and another setting	14 (14%)	[46,54,67,68,74,79,80,82,83,86,89,112,122,131]
	Healthcare setting	19 (18%)	[51,62,63,71,73,81,91,95,100,102-104,107,109,124,125,133,135,146]
	Research setting	12 (12%)	[52,59,87,98,110,115,118,127,128,132,140,142]
	Not reported	10 (10%)	[78,88,92,106,108,113,114,123,137,148]
**Supervision**	Supervised	32 (31%)	[47,52,57,62,63,70,71,73,78,81,83,87,88,92,98,102,104,108,113,115,118,120,123-125, 135,137,140-142,146,148]
	Both supervised and unsupervised	38 (37%)	[46,48-50,53,54,60,65-69,72,74,76,77,79,80,82,84,86,89-91,93,100,109,112,114,117,122, 129,130,131,136,138,144,145]
	Unsupervised		
	Not Reported	23 (22%)	[58,61,64,75,85,94-97,99,101,103,105,111,116,119,121,126,133,134,139,143,147]
		10 (10%)	[51,55,56,59,106,107,110,127,128,132]
**Supervision Contact**	Face-to-face (F2F)	48 (47%)	[46,47,52,57,60,62,63,67,68,71,73,78,81-84,86-89,91,92,98,100,102,104,108,109,112-115,117,118,120,122-125,135-137,140,142,144-146,148]
	Remote	12 (12%)	[48-50,65,66,69,72,74,93,129,138,141]
	Combination (F2F and remote)	10 (10%)	[53,54,70,76,77,79,80,90,130,131]
	Not supervised / unreported	33 (32%)	[51,55,56,58,59,61,64,75,85,94-97,99,101,103,105-107,110,111,116,119,121,126-128, 132-134,139,143,147]

**Numbers of DHT Types**	One<br><br>Two<br><br>Three	57 (55%)<br><br>30 (29%)<br><br>16 (16%)	[46,47,51-54,57,61,63,67,68,70,71,73,75-78,81,84-86,88-91,93,95,96,98,100,102-104, 106,109,112,114,115,123-126,129,130,132,133,135-138,140,143-147]<br>[48,49,55,56,58-60,62,64,69,83,92,94,97,99,101,105,107,108,110,111,113,116,118,121, 122,127,128,139,142]<br>[50,65,66,72,74,79, 80,82,87,117,119,120,131,134,141,148]
**DHT Type**	Virtual reality<br><br>App<br><br>Sensor<br>Activity monitor<br>Audio-video platform<br>Robotics<br>Messaging platform<br>Other (Brain computer interface technology, reminders on a wearable device and camcorder recording of sessions for remote review)	58 (56%)<br><br>31 (30%)<br><br>17 (17%)<br>16 (16%)<br>15 (15%)<br>13 (13%)<br>11 (11%)<br>4 (4%)	[46-49,51-53,57,59,62,63,65,66,69,71,72,74,77,78,80,81,84-89,92,95,96,98,102-104,106, 112,113,115,117-120,123-128,132,135-137,140,142,143,146-148]<br>[50,55,56,58,61,64-66,72,74,75,79,82,83,94,97,99,101,105,108,111,114,116,119-122, 131,134,139,141]<br>[50,55,56,87,94,99,101,105,108,110,111,116,119-121,134,141]<br>[67,68,73,76,79,80,82,91,100,109,122,129,138,139,144,145]<br>[48-50,54,65,66,69,70,72,74,79,80,93,131,141]<br>[59,62,87,92,107,110,113,117,118,127,128,142,148]<br>[58,60,64,82,83,90,97,117,130,131,134]<br>[60,107,133,148]

DHT: Digital health technology
