# Supplementary material for: Behavior Change Approaches in Digital Technology–Based Physical Rehabilitation Interventions Following Stroke: Scoping Review
Source: J Med Internet Res. 2024 Apr 24;26:e48725. doi: 10.2196/48725 (PMC11079774; doi:10.2196/48725)
Supplement: Multimedia Appendix 7 [file jmir_v26i1e48725_app7.pdf]

Multimedia Appendix 7. Behaviour change theories, models and frameworks reported (with associated references)

**Theory, Model or Framework**	**Studies (n=18), n(%)**	**Citations (as referenced in main manuscript)**
Social cognitive theory	6 (33%)	[76,109,111,121,129,130]
Behaviour change technique taxonomy	4 (22%)	[48,49,122,129]
Game design theory	3 (17%)	[47,57,125]
Operant conditioning	3 (17%)	[47,98,121]
Self-determination theory	3 (17%)	[48,49,126]
Arousal theories	2 (11%)	[70,113]
Goal setting theory	2 (11%)	[109,121]
Transtheoretical model	1 (6%)	[145]
Control Theory	1 (6%)	[129]
Intrinsic motivation theory	1 (6%)	[113]
Health action process approach	1 (6%)	[109]
Flow theory	1 (6%)	[102]
Persuasive technology	1 (6%)	[47]
