# Supplementary material for: Behavior Change Approaches in Digital Technology–Based Physical Rehabilitation Interventions Following Stroke: Scoping Review
Source: J Med Internet Res. 2024 Apr 24;26:e48725. doi: 10.2196/48725 (PMC11079774; doi:10.2196/48725)
Supplement: Multimedia Appendix 8 [file jmir_v26i1e48725_app8.pdf]

Multimedia Appendix 8. Individual behaviour change techniques coded

BCT Cluster	Total BCTs coded in cluster	Individual BCT	Total Individual BCTs coded
1. Goals and planning	85	1.1 Goal setting (behaviour)	14
		1.2 Problem solving	15
		1.3 Goal setting (outcome)	20
		1.4 Action planning	10
		1.5 Review behaviour goal(s)	7
		1.6 Discrepancy between current behaviour and goal	4
		1.7 Review outcome goal(s)	6
		1.8 Behavioural contract	7
		1.9 Commitment	2
2. Feedback and monitoring	119	2.1 Monitoring of behaviour by others without feedback	17
		2.2 Feedback on behaviour	83
		2.3 Self-monitoring of behaviour	12
		2.4 Self-monitoring of outcome(s) of behaviour	1
		2.5 Monitoring outcome(s) of behaviour by others without feedback	2
		2.6 Biofeedback	2
		2.7 Feedback on outcome(s) of behaviour	2
3. Social support	26	3.1 Social support (unspecified)	23
		3.2 Social support (practical)	2
		3.3 Social support (emotional)	1
4. Shaping knowledge	37	4.1 Instruction on how to perform a behaviour	34
		4.2 Information about antecedents	2
		4.3 Re-attribution	1

5. Natural consequences	3	5.1 Information about health consequences	1
		5.2 Salience of consequences	1
		5.3 Information about social and environmental consequences	1
6. Comparison of behaviour	23	6.1 Demonstration of the behaviour	17
		6.2 Social comparison	6
7. Associations	16	7.1 Prompts/cues	16
8. Repetition and substitution	6	8.1 Behavioural practice/ rehearsal	1
		8.7 Graded tasks	5
9. Comparison of outcome	2	9.1 Credible source	2
10. Reward and threat	57	10.1 Material incentive (behaviour)	1
		10.3 Non-specific reward	55
		10.4 Social reward	1
12. Antecedents	3	12.1 Restructuring the physical environment	1
		12.5 Adding objects to the environment	1
		12.6 Body changes	1
14. Scheduled consequences	5	14.1 Behaviour cost	1
		14.2 Punishment	2
		14.3 Remove reward	1
		14.10 Remove punishment	1
15. Self-belief	1	15.2 Mental rehearsal of successful performance	1

BCT codes which were not identified during intervention coding in this review have been excluded from the table.
