# Supplementary material for: Behavior Change Approaches in Digital Technology–Based Physical Rehabilitation Interventions Following Stroke: Scoping Review
Source: J Med Internet Res. 2024 Apr 24;26:e48725. doi: 10.2196/48725 (PMC11079774; doi:10.2196/48725)
Supplement: Multimedia Appendix 9 [file jmir_v26i1e48725_app9.pdf]

Multimedia Appendix 9. Behaviour change technique taxonomy clusters identified.

The pie chart shows the number of times a cluster was identified in the review (and percentage of the total number of clusters coded n=288). A cluster was only identified once per study, irrespective of the number of individual BCTs within that cluster.

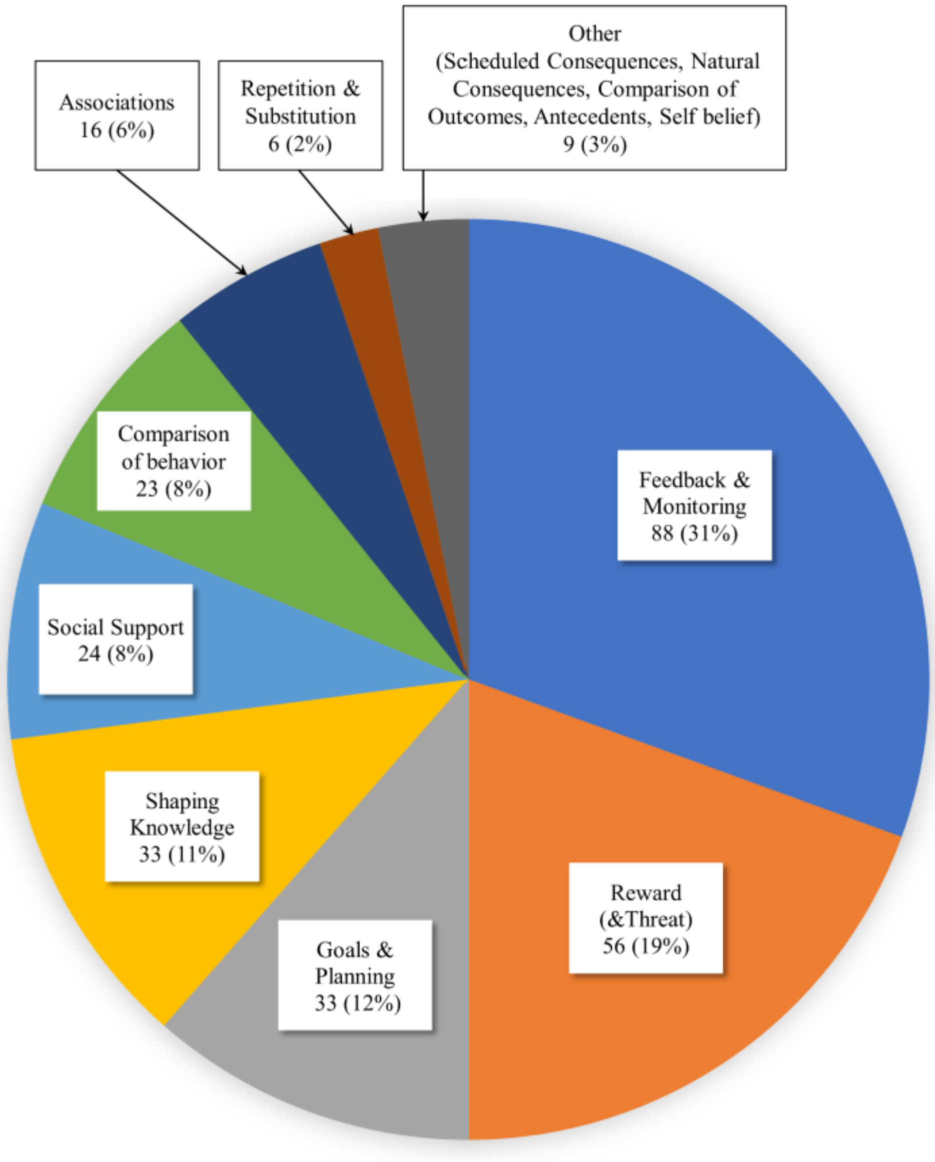

The table shows the number of studies (and percentage of the total number of studies n=103) in which a cluster was identified and the associated references.

**BCTTv1 Cluster**	**Studies (n=103), n(%)**	**Citations (as referenced in main manuscript)**
Feedback and Monitoring	88 (85%)	[46-60,62-69,71-74,76,78-80,82-92,94-106,108-113,116,117,119-129,134-146,148]
Reward and Threat	56 (54%)	[46-49,51-53,55-57,62,65,69,71,72,74,77,80,81,85,86,88,89,91,92,95,96,98,102,103,106-108,112,113,115,117-119,121-125,128,132,134-137,140,142,143,146-148]

Goals and Planning	33 (32%)	[49,58,60,65-68,70,72,74,76,79,80,82,83,90,91,93, 94,97,100,109,111,112,121,122,126,129,130,134, 138,141,145]
Shaping Knowledge	33 (32%)	[46,48,50,53-56,58,60,61,64-66,72,74,75,86,94,97, 101-103,108,111,113,114,120,129-131,139-141]
Social Support	24 (23%)	[48,49,58,60,64,67,70,72,73,79,80,82,84,90,93, 101,108,117,119,129-131,134,141]
Comparison of Behaviour	23 (22%)	[46,50,53,54,60,61,64-66,74,75,81,86,101,104,111, 114,118,122,123,125,131,139],
Associations	16 (15%)	[58,60,65,66,68,75,80,83,87,90,110,120,131,133, 139,144]
Repetition and Substitution	6 (6%)	[60,82,109,122,129,130]
Scheduled Consequences	3 (3%)	[47,80,88]
Natural Consequences	2 (2%)	[129,138]
Comparison of Outcomes	2 (2%)	[47,133]
Antecedents	1 (1%)	[60]
Self-Belief	1 (1%)	[70]
